# Clozapine-induced gastrointestinal hypomotility: UK pharmacovigilance reports, 2018–2022

**DOI:** 10.1192/bjo.2025.29

**Published:** 2025-03-31

**Authors:** Robert James Flanagan, Simon Alfred Handley, Charlotte James, Lilly Wells, Susanna Every-Palmer

**Affiliations:** Precision Medicine, Networked Services, King’s College Hospital, London, UK; Biochemistry, Department of Pathology, Royal Hobart Hospital, Hobart, Australia; Safety and Surveillance, Medicines and Healthcare products Regulatory Agency, London, UK; Department of Psychological Medicine, University of Otago, Wellington, New Zealand

**Keywords:** Clozapine, pharmacovigilance, gastrointestinal hypomotility, constipation, death

## Abstract

**Background:**

Clozapine-induced gastrointestinal hypomotility (CIGH) can cause constipation, which may progress to ileus, intestinal perforation and other life-threatening conditions. There were at least 527 unique cases of harmful CIGH (172 deaths) assessed by strict criteria in the UK, 1992–2017.

**Aims:**

To assess the impact of strengthened warnings about the risks of CIGH, such as those issued by the UK Medicines and Healthcare products Regulatory Agency (MHRA) (2017) and the US Food and Drug Administration (2020), on reports of harmful CIGH in the UK.

**Method:**

We audited UK MHRA Yellow Card reports recorded as clozapine-related gastrointestinal disorders, 2018–end 2022.

**Results:**

Of 335 unique reports (36 fatal, 26 male) that met initial CIGH criteria, there were 129 (22 fatal, 18 male) that met the final CIGH inclusion criteria. Reports of non-fatal CIGH (final criteria) averaged 26 per year (15 in 2022). Deaths averaged four per year (two in 2022). Where data were available the greatest proportion of deaths occurred after 10–14 years of clozapine treatment.

**Conclusions:**

Publicity aimed at raising awareness of the problem posed by CIGH has been associated with a reduction in harmful CIGH as reported to the UK MHRA since 2017. Continued vigilance is needed to reduce risk. Stopping smoking may pose a particular risk and should be monitored carefully.

In the UK some adverse drug reaction (ADR) data are obtained through reporting to the UK Medicines and Healthcare products Regulatory Agency (MHRA) Yellow Card scheme. In a study of 704 MHRA Yellow Card reports, 1992–2017, relating to the use of clozapine and coded as gastrointestinal disorders as per the Medical Dictionary for Regulatory Activities,^1^ 527 unique cases were assessed as harmful clozapine-induced gastrointestinal hypomotility (CIGH) according to strict criteria.^2^ There were 172 deaths in this CIGH cohort. In non-fatal CIGH, surgery was the most frequent outcome (*N* = 92). The data showed a general trend of increasing reports and deaths up until 2016. In 177 further people, CIGH likely contributed to the condition reported in many instances, but there was insufficient information to be sure.

## Potentially harmful CIGH

A complicating factor in attempting to collate data on the reported incidence of CIGH is that constipation is common in patients given clozapine,^3^ but defining the point at which constipation has progressed to potentially harmful CIGH is not straightforward. Of the 527 reports included in the above study, 1218 gastrointestinal ADRs were recorded (96 specific ADR terms). One patient had 10 different ADRs reported (abdominal distension, abdominal pain upper, diarrhoea, faecaloma, gastrointestinal motility disorder, paralytic ileus, intestinal dilatation, intestinal infarction, large intestinal obstruction and vomiting).

This difficulty in defining CIGH is likely the reason that systematic reports of CIGH only began to appear in 2008.^4^ This and subsequent publications^3^,^5^,^6^ led to the UK MHRA (2017) and the US Food and Drug Administration (FDA) (2020) issuing strengthened warnings about the risks of CIGH.^7^–^9^ There have also been recent ‘black box’ warnings in countries such as Australia.^10^

Pharmacovigilance is a dynamic process and the number of reports of reactions may alter following new information on hazards of medication becoming publicised. To assess the apparent impact of publicity aimed at minimising harm from CIGH, we have now studied all UK Yellow Card clozapine reports during 2018–2022 coded as gastrointestinal disorders to monitor trends in the prevalence and outcome of CIGH as reported to the MHRA over this period.

## Method

We audited reports to the MHRA Yellow Card scheme recorded as gastrointestinal disorders as per the Medical Dictionary for Regulatory Activities^1^ where clozapine was recorded as being either prescribed, or thought to be involved,^11^ 2018–end 2022 inclusive. The data were collated using a pre-specified data-extraction form.^2^ In summary, the information collected included patient demographics; ADR event characteristics such as date of onset and outcome; clozapine start date; clozapine dose at ADR onset; concomitant gastrointestinal ADR terms (in accordance with the World Health Organization (WHO) Adverse Reactions Terminology dictionary); clinical investigations; other medications; smoking habit; and any additional commentary.

A subset of reports forming the *initial* CIGH cohort was created where any of the following ADRs were noted: intestinal obstruction, paralytic ileus, small intestinal obstruction, intestinal perforation, intestinal ischaemia, intestinal pseudo-obstruction, ischaemic colitis, large intestinal obstruction, megacolon, ileus, large intestine perforation, gastrointestinal obstruction, intestinal infarction, colitis ischaemic, faecal vomiting, gastrointestinal necrosis, necrotising colitis, gastrointestinal perforation, gastric perforation, gastrointestinal ischaemia, gastrointestinal mucosal necrosis or ileal perforation. We also included reports where the following ADRs were recorded, but only when the outcome resulted in hospital admission or surgery, or was recorded as serious/life-threatening or fatal: constipation, faecaloma, oesophageal hypomotility, gastrointestinal hypomotility, functional gastrointestinal disorder, infrequent bowel movements, bowel movement irregularity, change of bowel habit, faecalith, faeces hard, gastrointestinal motility disorder, gastric hypomotility or impaired gastric emptying.

Each case was reviewed independently by two authors (S.A.H., S.E.-P.) to assess eligibility for inclusion in the *final* CIGH cohort. Reports where there were confounding comorbid conditions such as bowel cancer or inflammatory bowel disease that may have either caused or contributed to the gastrointestinal pathology were excluded. To eliminate duplication, multiple accounts of CIGH events for the same person (identified by demographic data and clinical details) were treated as single cases.

## Results

There were 295 unique reports that met the *initial* CIGH criteria (Table 1). Of these, 10 were excluded from further study because they were literature and not Yellow Card reports.^12–16^ In turn, there were 129 that met the *final* CIGH criteria. The corresponding data for 1992–2017 are included in Table 1 for comparative purposes.

**Table 1:** UK Medicines and Healthcare products Regulatory Agency Yellow Card reports, clozapine-induced gastrointestinal hypomotility (CIGH), 2018–2022 (% male in parentheses)

Year received	CIGH initial criteria		CIGH final criteria	
	Non-fatal	Fatal	Non-fatal	Fatal
*1992–2017*	*432 (68)*^a^	*276 (68)*^a^	*355 (72)*^a^	*172 (65)*^a^
2018	57 (53)	6 (83)	27 (41)	4 (75)
2019	54 (68)	7 (57)	25 (68)	5 (60)
2020	44 (66)	11 (91)	22 (59)	7 (100)
2021	56 (62)	6 (83)	18 (78)	4 (100)
2022	84 (64)	6 (33)	15 (80)	2 (50)
*2018–2022*	*295 (63)*	*36 (82)*	*107 (63)*	*22 (82)*

a . Data from Handley et al.^2^

**Fig. 1:**
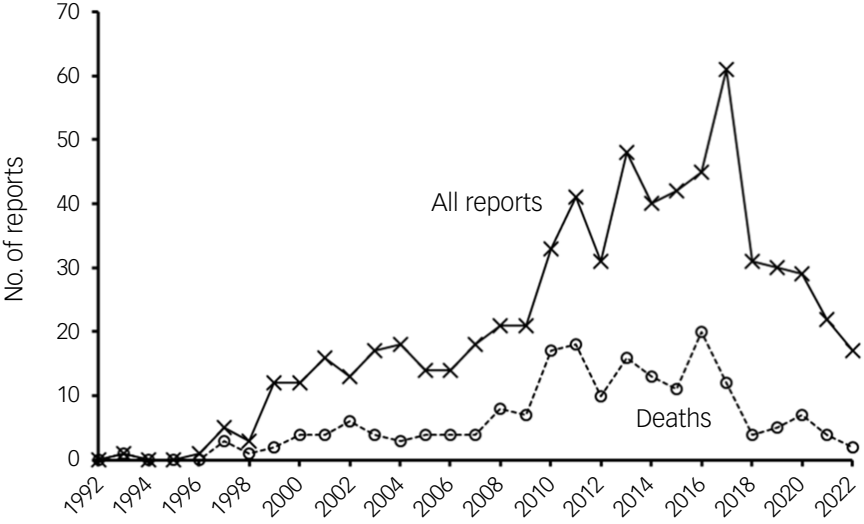
UK Medicines and Healthcare products Regulatory Agency Yellow Card reports, final clozapine-induced gastrointestinal hypomotility criteria, 1992–2022 (data for 1992–2017 from Handley et al^2^).

**Fig. 2:**
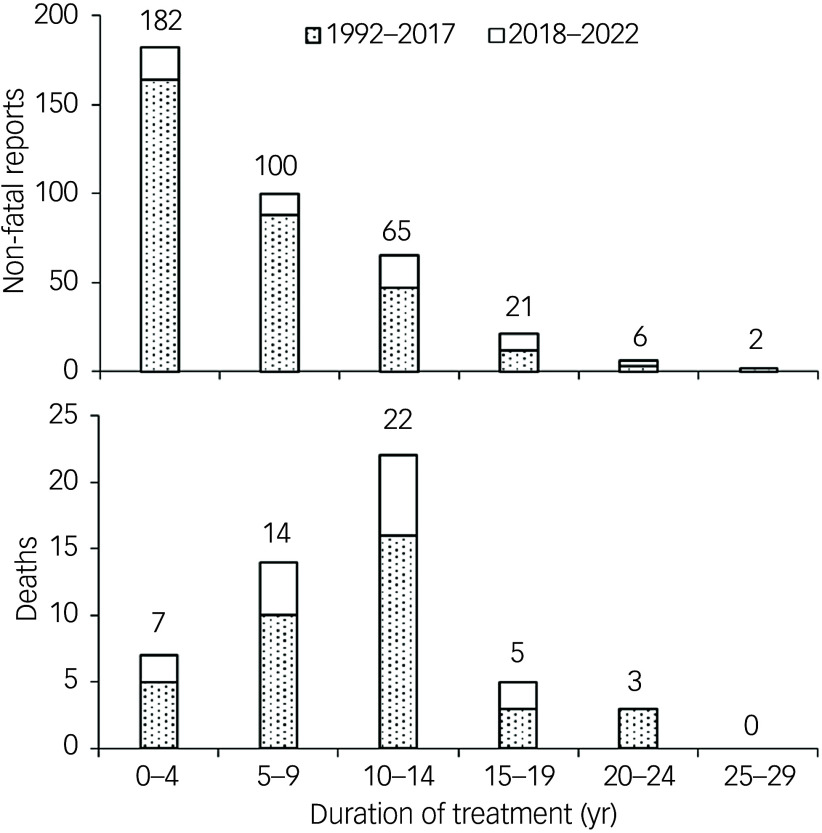
Duration of clozapine treatment until development of harmful clozapine-induced gastrointestinal hypomotility (*N* = 376 non-fatal, 51 fatal reports) (data from 1993 to 2017 from Handley et al^2^).

Reports of CIGH (final criteria) averaged four per year for 1992–2000, 16 per year for 2001–2008, 40 per year for 2009–2017, but only 26 per year for 2018–2022. Deaths averaged one per year for 1992–2000, five per year for 2001–2018, 14 per year for 2009–2017 and only four per year for 2018–2022 (Fig. 1). The duration of clozapine treatment until CIGH was recorded was provided for 376 (82%) and 51 (26%) non-fatal and fatal cases, respectively, for 1992–2022 (Fig. 2). Some 98 episodes of harmful CIGH occurred within the first year of treatment, but only two deaths were reported in this period. Within the first 4 years of clozapine treatment there were 191 reports, of which seven (3.7%) were fatal. At 10–14 years of clozapine treatment, there were 87 reports, of which 25% (*N* = 22) proved fatal.

In the years 2018–2022, there were 21 mentions of the use of laxatives in nine people before the onset of harmful CIGH in those who did not die. In the deaths, there were 21 mentions of the use of laxatives in two people before the onset of harmful CIGH. Opioids, tricyclic antidepressant drugs, other anticholinergics, antacids and antibiotics were mentioned infrequently. Likewise, smoking habit was rarely mentioned.

## Discussion

CIGH is a common and potentially serious adverse effect of clozapine. The CIGH spectrum ranges from dysphagia and constipation to megacolon, gastrointestinal obstruction and intestinal ischaemia. Death often ensues in severe cases.^2^ The data presented here (Table 1, Fig. 1) show that annual reports of harmful CIGH including deaths in the UK rose steadily up until 2016. Reports of harmful CIGH and deaths have since declined and, indeed, in 2022 reached the same level as in 2002. In this context, it should be noted that spontaneous pharmacovigilance reporting per se may be susceptible to selection bias, influenced by the seriousness and novelty of the effect, and by patient characteristics.^17^ It is thus important to stress that data collection has remained constant over time and is not spontaneous reporting as with most other drugs, but is especially rigorous thanks to the regulatory requirements surrounding the prescription and use of clozapine in the UK.

As noted by Handley et al,^2^ the move to non-smoking hospitals may have added to the problem posed by CIGH in the UK. Giving up smoking is itself associated with constipation in one in six smokers not taking clozapine, and for about one in 11 the problem can be severe.^18^ Further information comes from a study of 105 316/60 792 and 34 290/31 309 therapeutic drug monitoring (TDM) samples with dose information from male and female smokers/non-smokers, respectively. The data suggest that the optimal pre-dose plasma clozapine concentration may be different in smokers of either gender (0.35–0.45 mg/L) as opposed to non-smokers (0.50–0.60 mg/L).^19^ These data add to the suggestion that smoking may modify the effect of antipsychotics in ways other than by affecting CYP1A2 activity, possibly via an effect on the microbiome.^20^ Thus, although dose adjustment to minimise the risk of toxicity is required after smoking cessation,^21^ the resulting steady-plasma clozapine will likely be higher than when the patient was smoking, adding to the risk of CIGH.^22^

Treatment with clozapine decreases overall mortality in schizophrenia, in part by reducing suicidality.^23^ Clozapine may also limit substance misuse.^24^, ^25^ In terms of clozapine use, there were some 14 000 people prescribed clozapine in the UK in 2001 (Novartis, personal communication to R.J.F., 2001). In November 2019, the corresponding figure was 37 301.^26^ Thus, the increasing annual number of Yellow Card reports of potentially harmful CIGH up to 2016 may reflect in part the increased use of clozapine. It is tempting to speculate that the reduction seen since 2017 is the result of increased awareness of the risk posed by CIGH. The corollary is of course that the serious morbidity and mortality from CIGH seen since 2000 could likely have been reduced had the problem been better recognised 25 years ago.

Public attention and regulatory warnings have a significant effect on ADR reporting practice.^27^, ^28^ Unsurprisingly, as knowledge about an association between a drug and a serious adverse effect increases, so does the recognition and reporting of that ADR. However, deaths are less likely to be susceptible to such bias. In general, serious (and fatal) ADRs are reported more consistently.^29^ Furthermore, all patients prescribed clozapine in the UK must be registered with a supplier of the drug. Suppliers are informed of all clozapine de-registrations and the reason for the de-registration, and are obliged to report de-registrations, which of course include deaths, to the MHRA. This means the reduction in reports of harmful CIGH over the 5 years to 2022 likely reflects a true reduction in CIGH-related morbidity and mortality.

The results presented here summarise what is, to our knowledge, the longest pharmacoepidemiological study of CIGH, encompassing in all 31 years of data collection. This allows for comparison of trends over time, particularly in the context of recent increased awareness of the CIGH adverse effect spectrum. However, the Yellow Card reports studied here were only those in the gastrointestinal disorder category; hence, deaths coded as aspiration pneumonia or surgical procedure such as appendectomy, for example, without mention of gastrointestinal features as contributory factors will likely have been missed. This very diversity of clinical features makes it very difficult to define CIGH as discussed above. Indeed, it is likely that our strict CIGH inclusion criteria as described in the Methods section have underemphasised the scale of the problem in the UK.

### Clinical implications

Clozapine is thought to be some three times more likely to cause constipation than other antipsychotics.^3^ Higher pre-dose plasma clozapine concentrations (plasma clozapine concentrations are normally measured pre-dose) are associated with slower gastrointestinal transit.^22^ Thus, using the lowest effective dose, monitoring plasma clozapine concentrations and prompt dose adjustment if a patient stops smoking may help reduce the risk of harmful CIGH.

As to prophylaxis of CIGH, the use of pro-motility agents remains variable.^30^ Reports of severe complications of CIGH continue to appear.^31^ Prescribers should educate patients taking clozapine and their carers on the importance of not only reporting changes in smoking habit, but also in monitoring bowel function.^9^ It has been suggested that patients should be encouraged to maintain adequate hydration and fibre intake, and take regular exercise. However, evidence of the efficacy of these measures is lacking.

Hypersalivation was noted in 74.6% of clozapine-treated patients in a recent survey.^32^ There is much concern that concomitant use of anticholinergic drugs in an attempt to reduce salivation at night, in particular, may exacerbate CIGH. However, salivary hypersecretion was reported relatively infrequently in the CIGH cases studied by Handley et al.^2^ Concomitant use of anticholinergic drugs was likewise mentioned infrequently. This and the fact that harmful CIGH occurred after only 3 days of clozapine treatment^2^ and has been reported in a further patient recently^33^ suggests genetic susceptibility in certain people.

Careful history taking before commencing clozapine may help indicate patients at particular risk of CIGH. An imponderable is the absence of a simple test to detect the presence of potentially harmful CIGH analogous to the blood test used to detect neutropenia/agranulocytosis.^34^ Our view is that prophylactic laxative treatment should be started when clozapine is initiated as is done with opioids.^2^, ^35^ The systematic use of a laxative regime in clozapine-treated patients reduced the prevalence of serious CIGH from 8.2 cases per 100 person-years to 1.1 cases per 100 person-years (relative risk 0.13; 95% CI 0.403–0.043).^36^

Current recommendations for managing clozapine-induced constipation include use of osmotic agents such as polyethylene glycol and/or stimulants, for example senna or bisacodyl. An enema may be given *in extremis*. Bulk-forming or fibre-based laxatives are not recommended. Use of lubiprostone, a prostaglandin E_1_ analogue, together with agents such as docusate and lactulose may prove effective,^37^ as might the 5-HT_4_ receptor agonist prucalopride.^38^ In any event a collaborative approach involving not only the patient but also carers and primary care and mental health staff that emphasises both the hazard posed by CIGH and the need for continued adherence to laxative prophylaxis is vitally important.^39^

It has been suggested that use of fluvoxamine together with a lower dose of clozapine might be an effective combination in minimising the incidence of CIGH.^40^ However, fluvoxamine acts to delay clozapine elimination; hence, the amount of clozapine in the body associated with a therapeutic response to the drug would be the very similar if not the same as if a higher dose had been given without the addition of fluvoxamine. Therefore, this approach seems counter-intuitive. An alternative suggestion is to lower the dose of clozapine and, if necessary, add an antipsychotic with a lower propensity to cause anticholinergic effects.

### Regulatory implications

Clinical experience with clozapine in the UK now extends to some 35 years. Major concerns when granting the product licence were the risks of agranulocytosis when given chronically and of possibly fatal hypotension if given to a clozapine-naïve subject at the dose required for efficacy in schizophrenia. These led to the undoubtedly effective regular blood monitoring scheme and the ‘48-hour rule’, that is, the requirement to re-titrate the dose if doses had been missed for 2 days or more. Aspects of both of these requirements have now been questioned in the light of current knowledge.^41^, ^42^ An unintended consequence, however, has been the MHRA clozapine Yellow Card scheme, which is acknowledged to be amongst the best of its kind. Although CIGH was unsuspected in 1989, the Yellow Card and other data now show that laxative treatment must be considered when clozapine is instituted and bowel function monitored regularly whilst clozapine is continued to minimise the risk of progression to harmful CIGH. Data have also accumulated that emphasise other risks associated with clozapine therapy, such as pneumonia and venous thromboembolism.^43^

In summary, CIGH is a preventable cause of serious morbidity and mortality. Managing the risk posed by CIGH requires constant vigilance from all those involved in patient care.^9^, ^39^ Commencing laxative treatment when starting clozapine is an initial approach to minimising risk and is a way of introducing the patient and other carers to the importance of such therapy. Awareness of the risk of changes in smoking habit is also important. TDM may help ensure that the minimum effective dose is used. Research not only into better ways of monitoring bowel function in the absence of information from the patient but also into appropriate laxative treatment is still needed.

## Data Availability

The authors confirm that summaries of the data supporting the findings of this study are available within the article. The primary data are not publicly available because of patient confidentiality and can only be obtained by direct application to the MHRA.
